# The Impact of Emotional Solidarity on Residents’ Attitude and Tourism Development

**DOI:** 10.1371/journal.pone.0157624

**Published:** 2016-06-24

**Authors:** Ali Hasani, Sedigheh Moghavvemi, Amran Hamzah

**Affiliations:** 1 University of Science and Culture, Tehran, Iran; 2 Faculty of Business and Accountancy, University of Malaya, Kuala Lumpur, Malaysia; 3 Centre for Innovative Planning and Development (CIPD), Johor, Malaysia; Peking UIniversity, CHINA

## Abstract

In many countries, especially one such as Malaysia, tourism has become a key factor in economic development, and the industry heavily relies on feedback from local residents. It is essential to observe and examine the perceptions of residents towards tourists and tourism development for better planning in realizing successful and sustainable tourism development. Therefore, this research measured the relationship between residents’ welcoming nature, emotional closeness, and sympathetic understanding (emotional solidarity) towards tourists and their respective attitudes towards supporting tourism development. To test the proposed research model, we collected data using a questionnaire survey from 333 residents in rural areas in Malaysia. We used the structural equation modelling technique (Amos) to evaluate the research model, and the results revealed that the residents’ willingness (welcoming nature) to accept tourists is the strongest factor that effects the residents’ attitudes towards supporting tourism development. However, there was no significant relationship between residents’ emotional closeness and their sympathetic understanding towards tourists with their attitude and support towards tourism development. Welcoming nature, emotional closeness, and sympathetic understanding are able to predict 48% of residents’ attitudes towards tourism development and 62% of their support towards tourism development.

## Introduction

Over the past few decades, tourism has become one of the most rapidly growing economic sectors in several countries. This industry has experienced vivid growth and intensified diversification progress to emerge as a major contributor towards the overall economic advancement. Many tourists visit different destinations to partake in local customs, beliefs, history, cuisine, culture, and sports [1]. The tourism industry in Malaysia is growing expeditiously in a manner similar to other developing countries [2]. This fact is noticeable in terms of the contribution to foreign exchange earners, the growth of the Gross Domestic Product (GDP), employment opportunities [3], maximising of tax revenues, and diversifying economic opportunities to rural communities [4]. The Malaysian government is taking appropriate measures in promoting sustainable tourism [5]. To enhance the development of the tourism industry, the Malaysian government formed the Malaysian Tourism Policy in 1992. This policy arranged for all of its measures to promote ecotourism as a form of tourism that could be expanded and sustained [5]. Moreover, there were several other acts and policies that were formed and developed by the Malaysian government that favoured environmental issues [5], moving towards overcoming bureaucratic obstacles and a smoother implementation of sustainable tourism.

Although the government has plans to facilitate tourism development in the country, it must be understood that resident support and their attitudes towards tourism development are influential factors if those plans are to be successfully implemented. Those who live in tourist destinations could possibly experience positive (e.g., better job employment, interchange of culture) or negative impacts (e.g., increased cost of living, crowding), thus giving room to individuals to conceive attitudes about tourism development [6]. As a matter of fact, there is a link between residents and tourists, based on shared facilities and services within a destination [7, 8], and the utilisation of natural resources [9, 10].

Prior research has utilised theories/models such as the Social Exchange Theory (SET) [11, 12, 13, 14,15], the Social Representations Theory [16, 12, 17], Social Distance [18, 19], the Intimacy Theory [20, 21], the Integrative Theory of Cross-cultural Adaptation [22, 23], and the Contact-hypothesis Theory [24, 25, 26] to define the relationship between residents and tourists and to measure the cost and effect of tourism developments. They utilised many factors, such as socio-demographics (gender, education, age, length of residency and race/ethnicity), socio-economics (income and economic dependency), space (physical distance between residents and tourists), and travel behaviour (residents’ travel to local and abroad places) to analyse the residents’ attitude and perception about tourism.

Literature review shows that several researches have been conducted based on various factors that influence residents’ attitude and perception towards supporting tourism development. Most of these researches concentrated on specific dimensions of tourism development, and a few scrutinise the intricate feelings of residents towards tourists, which eventually affected their support for tourism development.

Taking into account the pool of potential and utilised variables, few have provided consistent support in explaining why residents perceive tourism and tourism development the way they do [12, 27]. Although there were several studies that defined the relationship between residents and tourists and despite the fact that many predicted residents’ attitudes towards tourism development [28], there was minimal research that addressed the closeness and intimacy of their relationship [29]. Also, frameworks that were utilised to describe residents’ attitudes were targeted mainly on tourism impacts and tourism development as a whole, and did not examine residents’ perception about tourists or what they have in common [29]. Woosnam [6] accurately pointed out that researchers failed to probe how the degree of emotional solidarity between residents and tourists can affect their respective attitudes towards tourism development.

Considering this gap, Woosnam and colleagues (2009 to 2011) investigated residents and tourists’ relationship to determine varying degrees of emotional solidarity with one another. They formulated items for emotional solidarity, shared beliefs, shared behaviour, and interactions between tourists and residents, and developed the scales for each of the four constructs [30]. The emotional solidarity scale concentrates on three elements, which are welcoming nature, emotional closeness, and sympathetic understanding factors. Examining emotional solidarity in the context of resident–tourist relationships, Woosnam [31] found that shared beliefs, shared behaviour, and interactions between members of each group significantly predicts individuals’ degree of emotional solidarity with each other. In 2012, Woosnam showed how emotional solidarity (three factors) significantly predicts two Tourism Impact Attitude Scale (TIAS) facets, which are support for tourism development and contributions tourism makes towards the community.

Considering the importance of the resident’s attitude towards tourism developments and interaction between tourists and residents and the aforementioned findings and literature gap, the main aim of this study is to measure the effects of a welcoming nature, emotional closeness, and sympathetic understanding on the resident’s attitudes and their support for tourism development in Malaysia. Particularly, this research will distinguish how the three factors are crucial towards influencing residents’ perceptions to support the tourism industry. This study used Woosnam’s model [6] as the research framework, and tested it in the context of Malaysia.

This research is vital because residents’ receptiveness towards tourists and tourism development serves as an important factor in the foundation of sustainable tourism, which refers to the combination of Quality of Life (QOL) for residents, prominent satisfaction level of tourists, and an appropriate environment platform for both residents and tourists. It is important to highlight the fact that tourism is not just about analysing the costs and benefits towards residents, but there is a necessity to delve deeper and dissect how residents’ personal feelings towards tourists determine their attitude towards tourism development and support, which may affect tourists satisfaction in the area. Moreover, since the Malaysian community is made up of different racial backgrounds with different cultures and beliefs, it is therefore of utmost importance to determine the community’s attitudes and examine their feelings towards support for tourism development [2].

## Literature Review and Hypothesis Development

Many tourism researches used Social Exchange Theory (SET) to explain the cost and benefits of tourism developments. Ap [32] describes SET as ‘‘a general sociological theory concerned with understanding the exchange of resources between individuals and groups in an interaction situation” (p. 668). In the tourism industry, SET is applicable when hosts and visitors exchange resources that are valued to both parties [33]. In fact, Sirakaya et al. [34]; Huttasin [35] suggested that resident attitudes toward tourism development rely on the recognition of perceived benefits and costs. Residents will normally engage themselves with tourists, thus forming a relationship that would maximise benefits and minimise costs [36, 37, 15, 27,34].

Investigations in the context of tourism revealed that a person who experiences benefits from an exchange would be more positive about tourism development, whereas ones that perceive costs are more likely to have negative perceptions about the tourism industry [38, 39, 40]. Thus, individuals who examine the exchange as beneficial recognise the same impact differently than someone who analyses the exchange as harmful [41]. Moreover, SET postulates that resident attitudes toward tourism development is influenced by their judgment of actual and perceived outcomes [38]. In this case, we can assume that some residents may be able to reap the benefits of tourism, while others may be negatively impacted. As an example, when host communities feel exploited by tourists, it will lead to a point of diminishing returns for them, thus negatively perceiving tourism as a whole [12]. Many researches in different countries used SET to measure the cost and effect of tourism developments.

Researchers such as Woosnam concentrated on other aspects of tourism developments and the relationship between residents and tourists, and argued that some residents and tourists participating in similar activities of common ground (shared behaviour) could cultivate greater cultural understanding and fortify their relationship [42]. Example of the shared behaviour are times when residents and tourists are seen shopping at local stores, or having a meal at local restaurants on a rural downtown community [7, 8], strolling on the beach, sightseeing and horseback riding [14], attending attractions such as museums and art galleries [43], participation in festivals and special events, outdoor recreation activities, and local patronage activities [44, 45, 16, 7, 42, 30]. Woosnam and Norman [30] argued that it is impossible to engage in shared behaviour without some degree of interaction between residents and tourists. For example, a realtor on Hilton Head Island enjoyed interacting with tourists by asking them where they are from and he also loved offering services and help them, while some residents had the opportunity to befriend tourists [42]. Indeed, some local residents feel good when tourists ask all sorts of questions about the destination, and when both parties get involved in the conversation, residents tend to feel closer towards tourists [42].

In 2009, Woosnam et al. conducted a qualitative research to develop the constructs of the theory of emotional solidarity based on the work of Durkheim [46] and investigate the relationship between destination residents and tourists’ and emotional solidarity. Durkheim [46] posits that the residents’ degree of shared beliefs, shared behaviour, and the level of interaction with tourists strongly predicts emotional solidarity. Hammarstrom [47] defined emotional solidarity as the effective bonds individuals experience with each other, characterised by perceived emotional closeness and the degree of contact. Jacobs and Allen [48] pointed out that emotional solidarity is a feeling of solidarity that binds a group together, fostering a sense of “we together” as opposed to a “me versus you” sentiment. Indeed, emotional solidarity does not necessarily denote the level of cooperation among individuals to achieve common ends [49], but it actually aims to strengthen individuals’ identity as part of a group [50].

Woosnam and Norman [30] developed and validated the scales for Durkheim’s theoretical framework of emotional solidarity and tested the model [31] to explain the relationship between residents and tourists. Woosnam suggested that shared beliefs, shared behaviour, and interaction are significant predictors of emotional solidarity (welcoming nature, emotional closeness, and sympathetic understanding) [31]. In fact, the sharing of similar beliefs and behaviours among residents and tourists and the positive interaction sessions will result in emotional closeness, sympathetic understanding, and welcoming, which will foster feelings of unity [31]. In 2012, Woosnam utilised the emotional solidarity factors to forecast levels of the Tourism Impact Attitude Scale (TIAS) and found a significant relationship between welcoming nature, emotional closeness, and sympathetic understanding towards residents support for tourism development and the contributions from tourism towards the local community.

**Fig 1:**
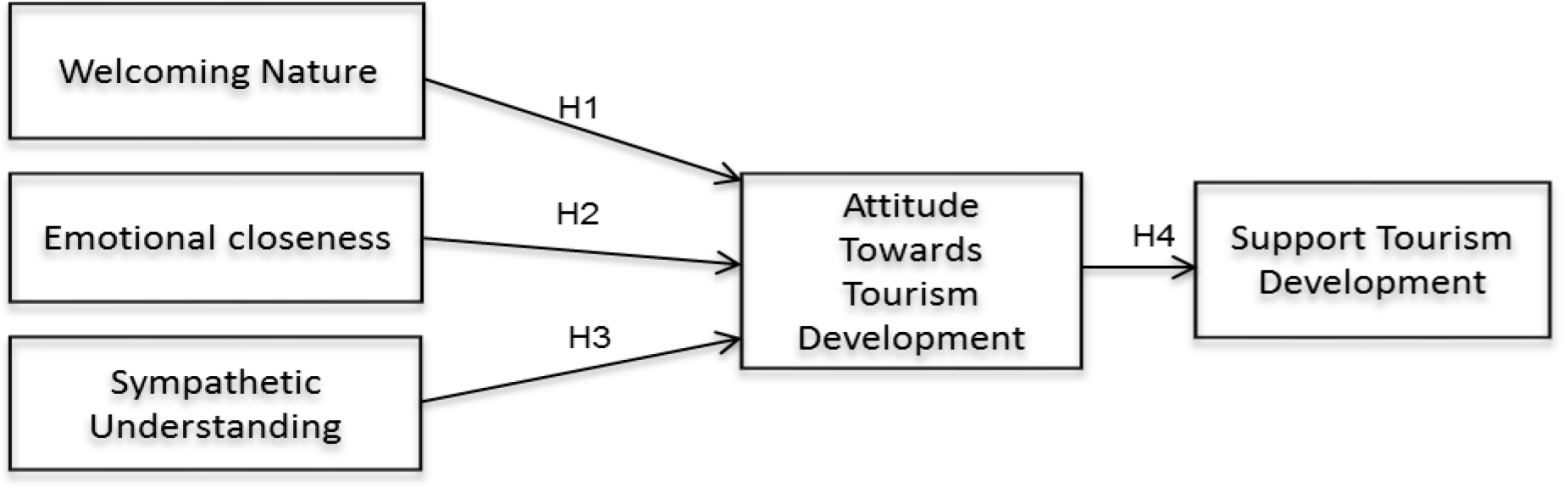
Research Framework.

Woosnam’s et al. [51] study probes the outcomes of the construct in the context of visitor expenditures among nature tourists. Out of the three ESS factors i.e. a welcoming nature, emotional closeness, and sympathetic understanding, welcomed feeling was the strongest. Moreover, these three factors were significant variables in the given model, and out of all these factors, the welcoming nature element was the best predictor, as it was significant in all of the models in the study. The current study used Woosnam [6] model, and took into account the emotional solidarity factors as predictors and its effect on resident’s attitude towards supporting tourism developments. The following sections will explain the welcoming nature, emotional closeness, sympathetic understanding (emotional solidarity), attitude, and support in detail for the development of the hypotheses (see Fig 1).

### Welcoming Nature

The “welcoming nature”, as Woosnam [6] argued, implies local residents’ personal interests in tourists and tourism. Woosnam [6] justified that a welcoming nature towards tourists significantly predicts the residents’ level of support for tourism development. Those residents who possess a welcoming nature toward tourists have bestowed personal interest in tourism development and duly benefited from it [52]. Thus, these residents have a positive attitude and will support tourism development within their respective communities [12, 53, 54,55]. In another context, Woosnam [29] deduced that residents tend to have a significantly higher degree of a welcoming nature compared to tourists, because residents regard it as their “home turf” and see themselves as hosts to the tourists [56]. Aramberr [57] strongly implied that a welcoming nature of local residents towards tourists would encourage them to learn from them, which would elevate tourism development initiatives. Accordingly, this study hypothesises that:

H1: Residents’ level of welcoming nature towards tourists will has a positive effect on their attitude towards tourism development.

### Emotional Closeness

Woosnam [6] discovered that emotional closeness with tourists does not directly predict the residents’ level of support for tourism development. Woosnam [6] mentioned that emotional closeness strongly predicts the contributions the tourism industry provides to its community. However, residents who developed and shaped emotional closeness with tourists have forged friendships with them, and are better equipped to recognise the contributions of tourism development to the community [6]. Such friendships are a paramount example of the social impacts reaped via tourism in a destination [6]. Based on Woosnam and Aleshinloye’s [58], it has been deduced that if residents have greater and more positive interaction sessions with tourists, then it is more likely that residents may perceive a sense of closeness with tourists. On the other hand, when residents start interacting with tourists, this would diminish the hostility and prejudices [59], thus elevating emotional closeness [29]. The emotional solidarity theory states that the commonalities between tourists and residents could possibly develop into relationships and emotional affinity [60]. If residents perceive commonalities with tourists, “this reflects the residents’ willingness to realise they are not entirely different from tourists, debunking the standard ‘us’ versus ‘them’ mind-set” [42]. Accordingly, this study hypothesises that:

H2: Residents’ level of emotional closeness towards tourists will has a positive effect on their attitude towards tourism development.

### Sympathetic Understanding

Woosnam [61] pointed out that sympathetic understanding towards tourists significantly predicted the residents’ level of support for tourism development. It can be assumed that if residents have higher levels of sympathetic understanding towards tourists, they would have a more positive and supportive attitude towards tourism development [6]. Moreover, Woosnam [6] also justified that sympathetic understanding had a strong influence on attitude. Principally, if these people reside in a tourist destination area, they will be able to interpret and apprehend the feelings of residents and tourists [62]. Many residents and tourists posited that they experienced a positive degree of sympathetic understanding with one another because residents are accustomed to the fact that tourists utilise amenities at their destinations [31]. This kind of empathy shown by residents gives them an opportunity to “put themselves in others’ shoes”, as far as the negative social impacts of tourism in their community is concerned [63]. Following the above rationale, this study hypothesises that:

H3: Residents’ level of sympathetic understanding towards tourists will has a positive effect on their attitude towards tourism development.

### Residents’ Attitude toward Tourism Development

The topic of the residents’ attitudes towards tourism has been a well-studied subject in the area of tourism industry, and researches have incorporated studies on the attitude and perception of residents towards tourism support and development [27]. Kotler et al. [64] defined attitude as an element that describes a person’s relatively consistent evaluations, feelings, and tendencies towards an object or an idea. In the tourism context, attitudes and perceptions are actually derived from the perceived tourism-related benefits and costs experienced by the residents [65]. Research shows that the residents’ attitudes influence tourists’ satisfaction levels and repeat visitation to the host community area [56]. In short, residents would incorporate positive attitudes toward the tourism industry if they presume that the industry would foster job creation, provide better incomes, and provide opportunity to meet new and interesting people while improving facilities and infrastructures [66].

There may be some residents who would welcome tourists with open arms, while there would be others who view tourists with ambivalence. Moreover, residents’ attitude towards tourism evolution can be described in the context of socioeconomic, socio-demographics, and travel behaviour variables [6]. When residents become economically dependent on the tourism industry [67, 27] and experience its benefits and gains [53, 54,55], they would be more likely to support its development. Based on Perdue et al [39] and Látková and Vogt’s [68], the residents’ attitudes towards tourism development have been classified into two main categories, which are support for additional tourism development and support for restrictions on tourism development. Therefore, this study hypothesises that:

H4: Residents’ attitude towards tourism development will has a significant effect on their support on tourism development.

Literature review shows that a large number of studies have been conducted based on various factors that influence residents’ attitudes and perceptions towards supporting tourism development. Most of this research concentrated on specific dimensions of tourism development. Among all these prior studies, only a few scrutinise the intricate feelings of residents towards tourists, which eventually affected their attitudes towards the support for tourism development.

## Method

### Participants and procedures

The sampling frames used in this study are residents living in two tourist’s destinations, Pahang and Sabah in Malaysia. Pahang is the third largest state in Malaysia, after Sabah and Sarawak. Based on the published tourist arrival statistics, the number of tourists heading to Pahang from January to December 2015 were 996,004 international visitors, 1,459,705 ASEAN visitors, and 8,078,002 domestic visitors [69]. Sabah is one of the thirteen states and the second largest state in Malaysia, and it shares the Borneo Island with Brunei, Indonesia, and Sarawak. Based on the tourism statistics of 2016, there were a total of 97,388 international visitors and 156,499 local visitors who visited Sabah in the month of January 2016 [70]. Moreover, based on similar statistics, there were a total of 101,277 international visitors and 176,421 Malaysian visitors who visited Sabah in February 2016 [71]. For the current study, the survey was distributed to 1,000 residents older than 20: the first was based on judgement sampling, where we selected two tourist destinations (Pahang and Sabah), which has many international and local tourists, and distributed the questionnaire personally to the respondents based on convenience sampling. We collected 340 questionnaires, -with a response rate of 33%- of which only 333 were usable in this study (seven questionnaire was not completed and deleted from the study), which is adequate for this study (5 or 10 cases per parameters), [72] for use in structural equation modelling (Amos). This study did not collect any information related to the name and identity of the respondents, and the data was aggregated and were analysed anonymously. Of these respondents, 43.2% were men and 56.2% were women. The average age of the respondents was ~35 years. Most (90%) are Muslims, with 11–30 years old residency. A total of 68.5% are Malay, 3.1% are Chinese, and 0.3% are Indians, while 28% are other ethnic group.

### Measurement

The foundation of this research is based on Woosnam’s [6] framework that examines a welcoming nature (4 items), emotional closeness (4 items), and sympathetic understanding (3 items), and their effects on residents’ attitudes (6 items) towards the support (6 items) for tourism development (S1 Appendix). This study tested the model in rural areas in Malaysia to explore the welcoming nature, feelings, and sympathetic understanding of residents in the country towards tourists and tourism developments. We modified some items to better fit the current research context. Likert scales (1–7) ranging from ‘strongly disagree’ to ‘strongly agree’ were used for all the construct items, and residents were asked to rate their perception towards tourists and support for tourism developments within that area.

## Results

We tested our model using structural equation modelling, Amos 18. In this study, the exploratory factor analysis in SPSS using the principle component method with varimax rotation was performed to verify whether the questionnaire items were properly mapped to the corresponding construct. The Cronbach’s alpha coefficient for all dimensions exceeded 0.811, which represented an acceptable level for construct validity, indicating a high content consistency between the questions related to each of the constructs (S1 Appendix). The results of examining the correlations among the independent and dependent variables indicated that all the values are within the acceptable range (0.4–0.7). Based on Pallant [73], values of more than 0.8 or 0.9 are perhaps reason for concern. We conducted a confirmatory factor analysis through AMOS to test the measurement model and explain how the measured variables logically and systematically represented the constructs involved in a theoretical model. The results of the assessment for model fit indices showed four of the goodness-of-fit indices. The Chi-square (χ2) value is 513.65, the χ2 /df has a value of 2.675, the Comparative Fit Index (CFI) has a value of 0.930, and the Root Mean Square Error of Approximation (RMSEA) has a value of 0.071, which are all regarded as acceptable. However, three other assessments of goodness-of-fit indices, including the Tucker Lewis Index (TLI) with a value of .915, goodness of fit (GFI) value of 0.882, and Adjusted Goodness of Fit Index value of 0.845 are close to the acceptable value of 0.90 yield adequate model fit for the empirical data [74].

We used composite reliability and Average Variance Extracted (AVE) to evaluate convergent validity. The composite reliabilities of all the five constructs exceeded 0.8. The Average Variance Extracted (AVE) for each construct was greater than 0.50 (Table 1). The results of these tests provided evidence for the convergent validity [74]. Furthermore, we examined the factor analysis results and compared AVEs with the inter-construct correlations to evaluate discriminant validity. The evaluation of the structural model was then able to commence. The Structural Equation Modelling (SEM) technique was used to test a set of relationships between the independent and the dependent variables. The results of the structural model showed that the model achieved a good level of fit, χ2 = 542.003; χ 2 / df = 2.823; GFI = 0.876; AGFI = 0.836; TLI = 0.908; CFI = 0.923; RMSEA = 0.074.

**Table 1:** Descriptive Statistics, Correlation, Composite Reliability and Average Variance Extracted.

	Mean	SD	CR	AVE	1	2	3	4	5
1. welcoming nature	4.25	0.66	.829	.739	**0.859**				
2. emotional closeness	4.10	0.69	.818	.727	0.628**	**0.852**			
3. sympathetic understanding	3.86	0.73	.759	.711	0.487**	0.712**	**0.843**		
4. residents’ attitude	4.21	0.66	.901	.773	0.554**	0.503**	0.400**	**0.879**	
5. support for tourism development	4.16	0.811	.872	.949	0.503**	0.453**	0.417**	0.573**	**0. 974**

**p< .01

CR = Composite reliability, Value on diagonal are square root of AVE

The results (Table 2) indicated that the effect of residents’ welcoming nature towards attitude is strong, positive, and significant, while the effect of emotional closeness and sympathetic understanding towards residents’ attitude to support tourism development is not significant. This indicates that H1 was supported, while H2 and H3 were not. The results showed that there was a strong and positive relationship among resident’s attitudes and support for tourism developments, which supported H4.

**Table 2:** Hypothesis testing on residents support for tourism development.

Hypotheses				β	S.E.	C.R.	P Support	
H1	Welcoming Nature	→	Attitude	0.547	0.112	5.746	***	**Yes**
H2	Emotional Closeness	→	Attitude	0.149	0.159	0.810	0.418	No
H3	Sympathetic Understanding	→	Attitude	0.042	0.120	0.259	0.795	No
H4	Attitude	→	Support Tourism Development	0.788	0.083	10.570	***	**Yes**

β: Standardized Regression Weight S.E.: Standardized Error; C.R.: Critical Ratio;

***p< 0.001

## Discussion

The main objective of this study is to measure the welcoming nature, emotional closeness, and sympathetic understanding among residents and tourists and their respective effects on the residents’ attitudes towards support for tourism developments.

The findings showed that there is a significant relationship between a welcoming nature and the residents’ attitudes. This supports hypothesis 1 *(H1)*, which states that the residents’ level of welcoming nature towards tourists will affect their attitude towards supporting tourism development. The welcoming nature is related to the personal interests of residents towards tourism development and the presence of tourists in the area. The “welcoming nature”, as Woosnam [6] put it, implies local residents’ personal interests in tourists and tourism. Therefore, it can be justified that Malaysian local residents are proud and happy to welcome international tourists and foreigners to their country. Moreover, it can be presumed that they appreciate the contributions made by tourists to their community and country, thus turning towards a more positive attitude vis-à-vis tourism development. When the welcoming nature is present, it can be assumed that residents and tourists tend to share a certain amount of similar beliefs and behaviours, and they surely interact positively with one another, resulting in a better level of emotional closeness that fosters unity [29]. Indeed, as justified by Woosnam [6], the welcoming nature could significantly and positively predict local residents’ attitudes and support towards tourism development. Woosnam et al [51] denoted that a welcoming nature is deemed the best predictor of the residents’ attitudes towards tourism development.

On the other hand, this study found that there was a non-significant relationship between emotional closeness and sympathetic understanding with residents’ attitudes. Therefore, this does not support hypothesis 2 (H2) and hypothesis 3 (H3), which means that residents’ level of emotional closeness and sympathetic understanding towards tourists did not affect their support of tourism development. This showed that tourists and residents are not emotionally connected, although they benefit from each other in different ways. This finding is consistent with Woosnam’s [6], who found that emotional closeness with tourists does not directly predict the residents’ level of support for tourism development. Moreover, Wang and Xu [75] suggested that the residents’ emotional feelings towards tourists may not be spontaneous, as this factor will strongly rely on their self-concept towards tourists’ travel behaviour and attitudes (how these two factors contribute to the community). Sometimes, when there are dissimilarities in language or if there is a language barrier, it reduces emotional closeness between residents and tourists [76]. However, this does not mean that residents do not support tourism development. Moreover, residents very frequently speak in their native language(s), making it difficult to communicate and connect with the tourists. The residents’ level of emotional closeness sometimes depends on the level of interest tourists have in the local culture of that destination, which would determine the receptivity level of them easily befriending residents [77]. Wearing et al. [78] pointed out that emotional closeness also depends on the friendly nature of residents and the contentment of tourists in getting to know the locals.

The fact that sympathetic understanding did not affect residents’ attitude towards tourism development contradicted Woosnam’s [6], who posited the exact opposite. This could be explained by the fact that residents lack commonalities with tourists for them to engage at a certain level of understanding [29]. Alternatively, the tourists’ duration of stay, on average, was normally a few days, and this time constraint could have been a contributing factor for residents not being able to engage in interpersonal relationships with tourists and not being able to share much of their similar beliefs and behaviour with tourists. Due to this disadvantage, it is assumed that the level of interaction between residents and tourists could be negatively affected as well. Principally, if residents stay beyond the tourist destination area, there could be a possibility that they would not have the chance to interpret and apprehend the feelings of the tourists [62].

From this research, there is a significant relationship between residents’ attitudes and their support for tourism development. This supports hypothesis 4 (H4), where residents’ attitudes towards tourism development has a positive effect on their support of tourism development. This hypothesis could be justified because Lepp [66] suggested that residents’ attitudes towards tourism is a barometer to determine their support for tourism development. Based on the Theory of Planned Behavior (TPB), attitude towards a certain behaviour (in this case it is support for tourism development) is determined through a series of assessments of one’s beliefs based on the consequences that could arise from one’s behaviour and an evaluation of the desirability consequences [79]. Alternatively, when it comes to the Social Exchange Theory (SET), this theory states that residents engage in an exchange process once they have realised the rewards and costs, and residents will only enter into a relationship with tourists if they can maximise benefits and minimise costs [80]. Therefore, it is proven in this study that residents who benefited from the tourism industry have a positive attitude towards tourism advancement, thus supporting tourism development as a whole [80].

### Theoretical and practical implications

The foundation of this study is based on Woosnam’s [6] model of emotional solidarity, which incorporated how welcoming nature, emotional closeness, and sympathetic understanding influenced residents attitude, and their support towards tourism development in the Malaysian context. This study validated Woosnam’s [6] model in a new context and shows that the results may not be similar in different cultural settings. This research validated Woosnam’s [6] research framework in the context of Malaysia and allows readers to comprehend emotional solidarity and how it explains the level residents treat tourists, the level of residents’ personal feelings and closeness towards tourists, and how much residents have in common with tourists in different cultures and contexts. The results of this research support Woosnam’s [6] findings that the relationship between residents and tourist is more than cost and benefits, and researchers should investigate other aspects, such as the link between residents and tourist and emotional solidarity for sustainable tourism. Since this study is conducted in the Malaysian context, the outcome from this research would be advantageous to government authorities in this country, as they would be able to understand the needs of their local residents so that they would not be affected by the potential severe negative impacts of tourism development. As a matter of fact, it is vital to apprehend residents’ passion and fondness towards tourists, as this is the foundation of realising sustainable tourism. This model can tested at different tourist destination in the country, and based on the results, tourism planning entities can take action to improve the level of emotional solidarity among tourist and residents via different methods such as hosting different workshop to increase local awareness about tourists, improve homestay program, hosting cultural and local events, which will increase the interaction between tourists and residents and emotional solidarity.

### Research limitation, and future research

Although this study had contributed helpful information, there were some pervasive limitations. Since the sampling method used in this study was judgement sampling (based on tourist arrival), there would be a high probability that the group selected as respondents may not be representative of the entire population, since Malaysia has multiple tourist destinations. Future research should collect data from these other locations in Malaysia. In this research, there was less opportunity to examine the reactions of residents from different areas and states in Malaysia. Future research could also collect data from tourists (international and local) and residents at similar destinations and compare the results. Moreover, instead of just focusing on the three elements of emotional solidarity, it would be ideal if emotional solidarity is combined with other impacts conceived by the tourism industry to examine residents’ attitude and level of supportiveness towards tourism development in order to get a an absolute understanding of residents’ perception, attitude, and reaction towards the industry.

## Conclusion

This study examined the Emotional Solidarity Scale (ESS) model [6] in the rural areas of Malaysia. This research specifically measured the effects of the residents’ welcoming nature, emotional closeness, and sympathetic understanding towards tourists, which ultimately influenced their attitudes towards support for tourism advancement. The finding supported the model and showed that a welcoming nature is the only factor that has a strong effect on the residents’ attitudes and support for tourism development. This implies that the residents appreciated the benefits obtained from the tourists by their community, and appreciate the fact that foreign visitors contribute towards the local economy, which agrees with Woosnam’s et al [42]. The non-significant effect of emotional closeness and sympathetic understanding between tourists and residents on the residents’ attitudes toward tourism development highlighted the fact that this level of connection is more suitable for examination among repeat tourists who have visited the area many times and developed a deep psychological attachment, which future research can consider. This study is vital because it allows us to analyse the effects of these factors, as tourism has become a key development initiative in many countries, and prosperity in this industry partly relies on responses from local residents. It is essential to observe and examine the behaviour pattern of residents towards tourists, because if this study does not follow through, then it would be a challenge to attain sustainability in the tourism industry. The result of this study is useful for the industry players and policy makers planning on attracting tourists to their countries.

## Supporting Information

S1 Appendix (DOCX)

S1 Dataset (SAV)
