# Supplementary material for: The Impact of Emotional Solidarity on Residents’ Attitude and Tourism Development
Source: PLoS One. 2016 Jun 24;11(6):e0157624. doi: 10.1371/journal.pone.0157624 (PMC4920400; doi:10.1371/journal.pone.0157624)
Supplement: S1 Appendix — (DOCX) [file pone.0157624.s001.docx]

**Appendix A**

| No. | Variables (Items) | Cronbach α |
| --- | --- | --- |
| 1 | **Welcoming Nature**  I am proud to have tourists in this area/community  I feel the community benefits from having tourists in this area  I appreciate tourists for the contribution they make to the local economy  I treat tourists well in this area/community | 0.837 |
| 2 | **Emotional Closeness**  I feel close to some tourists I met in this area/community  I have made friends with some tourists in this area/community  I enjoy interacting with tourists  My interactions with tourists are positive and useful | 0.831 |
| 3 | **Sympathetic Understanding**  I have a lot in common with the tourists in this area/community  I feel affection towards tourists in this area/community  I understand tourists in this area/community | 0.744 |
| 4 | **Attitude**  Having tourists in the area/community is a good idea  Improving tourism activity in the area/community is a wise idea  I like the idea of having more tourists in the area/community  Having tourists in this area/community is pleasant  The presence of tourists in this area/ community is interesting  Having tourists in this area/ community is fun | 0.908 |
| 5 | **Support**  Tourism can be one of the most important industries for a community  I support the development of community based sustainable tourism initiatives  I support new tourism facilities that will attract new tourists to my area/community  Additional tourism would help this community grow in the right direction  I am happy and proud to see tourists are interested with what my community has to offer  I believe tourism should be actively encouraged in my area/community | 0.871 |
